# Gender differences in colorectal cancer: implications for age at initiation of screening

**DOI:** 10.1038/sj.bjc.6603628

**Published:** 2007-02-20

**Authors:** H Brenner, M Hoffmeister, V Arndt, U Haug

**Affiliations:** 1 Division of Clinical Epidemiology and Aging Research, German Cancer Research Center, Bergheimer Strasse 20, D-69115 Heidelberg, Germany

**Keywords:** colonoscopy, colorectal cancer, prevention, sigmoidoscopy

## Abstract

There is some variation regarding age at initiation of screening for colorectal cancer (CRC) between countries, but the same age of initiation is generally recommended for women and men within countries, despite important gender differences in the epidemiology of CRC. We have explored whether, and to what extent, these differences would be relevant regarding age at initiation of CRC screening. Using population-based cancer registry data from the US and national mortality statistics from different countries, we looked at cumulative 10-year incidence and mortality of CRC reached among men at ages 50, 55, and 60, and found that women mainly reached equivalent levels when 4 to 8 years older. The gender differences were remarkably constant across populations and over time. These patterns suggest that gender differentiation of age at initiation may be worthwhile to utilise CRC-screening resources more efficiently.

With more than one million new cases and more than 500 000 deaths per year, colorectal cancer (CRC) is the third commonest cancer and the fourth commonest cancer cause of death worldwide (Parkin *et al*, 2005). Owing to its typically slow development, there is a large potential for reducing the burden of the disease by early detection and removal of precancerous lesions or early cancer stages. Various screening examinations, including faecal occult blood testing (FOBT), sigmoidoscopy, and colonoscopy have meanwhile been recommended by expert committees and implemented in screening offers in a number of countries (e.g. Winawer *et al*, 2003; Schmiegel *et al*, 2004; Malila *et al*, 2005; Smith *et al*, 2006). Regarding the age at initiation of screening, which is a crucial parameter for the effectiveness and cost-effectiveness of screening programmes (Vijan *et al*, 2001), there is some variation between countries (typically ranging from 50 to 60 years for the population at average risk). However, within countries, the same age of initiation is generally recommended for women and men, despite important gender differences in the epidemiology of CRC. In particular, age-specific CRC incidence and mortality are lower in women than in men, which implies that women reach comparable levels of CRC incidence and mortality at higher ages than men. This paper aims to address the question whether and to what extent these epidemiological differences might be relevant for defining age at initiation of CRC screening among women and men.

## METHODS

### Outcome measure

When attempting to translate the gender differences in the epidemiology of CRC into gender-specific ages at initiation of screening, it is first necessary to define an appropriate outcome measure. In a screening programme that primarily aims at the early detection of CRC, as applies to FOBT-based screening, (cumulative) age- and sex-specific CRC incidence appears to be a natural choice. In a screening programme that aims to detect both CRC and its precursors (adenomas), as applies to endoscopy-based screening, the epidemiology of adenomas might also be considered. However, if the time period until adenomas progress to cancer is independent of age and sex, additional consideration of adenoma occurrence would have no impact on the results. If, on the other hand, progression time differs by age and sex, gender-specific differences in CRC incidence may actually remain the more relevant parameter even for these types of CRC screening programmes. When data on CRC incidence are not available, age- and sex-specific CRC mortality can be used as a good surrogate parameter, given that survival differences between female and male CRC patients are very small (Bossard *et al*, 2007).

### Data sources

Age and sex specific data on CRC incidence and mortality were obtained for the years 2000–2003 in the US from the National Cancer Institute's Surveillance, Epidemiology and End Results (SEER) Programme (Ries *et al*, 2006). The incidence data are based on 17 areas of the US (Atlanta, Connecticut, Detroit, Hawaii, Iowa, New Mexico, San Francisco-Oakland, Seattle-Puget Sound, Utah, Los Angeles, San Jose-Monterey, Rural Georgia, Alaska, Greater California, Kentucky, Louisiana, and New Jersey). The mortality data are based on US national figures.

In addition, the World Health Organisation (WHO) mortality database, accessed through the website of the International Agency for Research on Cancer (WHO mortality data base, 2006), was used to assess the consistency of observed patterns between populations. National age- and sex-specific mortality data referring to the year 2001 were obtained for 11 large countries from different parts of the world: Australia, Asia (Russia, Japan), Europe (France, Germany, Italy, Poland, Spain, United Kingdom), and North America (Canada, US). Although data up to the year 2003 were available for some countries, the latest available data were from 2001 for other countries; we therefore chose this year for our comparative analyses. Finally, US national mortality data from the years 1976, 1981, 1986, 1991, 1996, and 2001 were obtained from the WHO-mortality database to address consistency of observed patterns over time.

### Statistical analysis

Using the SEER incidence data, we first calculated cumulative CRC incidence within the following 10 years among men and women for each single year of age between ages 50 and 75. The cumulative incidence within a given age range approximates the expected risk of developing a disease within the defined age interval in the absence of competing causes of death, assuming that age specific incidence rates remain constant over time (Day, 1987). Starting from the levels of 10-year cumulative incidence among men at ages 50, 55, and 60 (the most commonly implemented ages for initiation of CRC screening in existing programmes), we determined at what ages the same levels of 10-year cumulative incidence were observed among women. Analogous calculations of ‘risk advancement periods’ (Brenner *et al*, 1993) were then carried out for 10-year cumulative mortality on the basis of the national vital statistics data. In additional sensitivity analyses, the time interval was varied between 5 and 15 years.

## RESULTS

**Figure 1:**
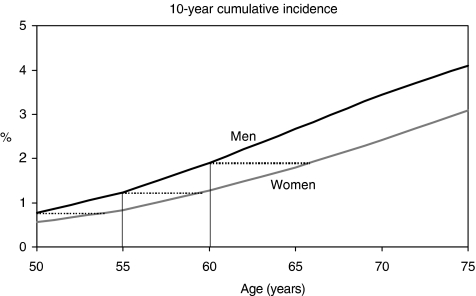
10-year cumulative incidence of colorectal cancer in subsequent 10 years among men and women at various ages. The dotted lines indicate the age differences at comparable levels of cumulative incidence between women and men. SEER Program, US, 17 registries, 2000–2003 (Ries *et al*, 2006).

The SEER incidence database included 74 111 men and 72 290 women diagnosed with CRC in 2000–2003. Among men, cumulative incidence in the subsequent 10 years increased from 0.8% at age 50 to 1.2% at age 55 and 1.9% at age 60 (see Figure 1). Among women, comparable levels of 10-year cumulative incidence were reached at ages 54, 60, and 66 only, i.e. 4, 5, and 6 years later, respectively.

**Figure 2:**
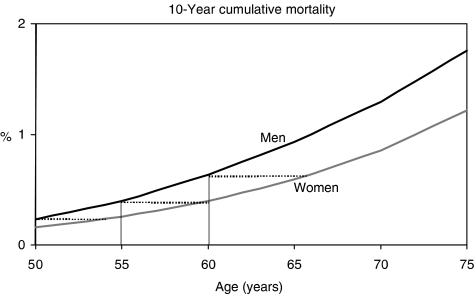
10-year cumulative mortality from CRC in subsequent 10 years among men and women at various ages. The dotted lines indicate the age differences at comparable levels of cumulative mortality between women and men. US national mortality statistics, 2000–2003 (Ries *et al*, 2006).

Overall, 113 174 men and 113 454 women died of CRC in the US in 2000–2003. The 10-year cumulative mortality from CRC in the subsequent 10 years also steadily increased with age in both sexes, and it was higher at any age between 50 and 75 years among men than among women (see Figure 2). Cumulative mortality within the subsequent 10 years was 0.23, 0.39 and 0.63% at ages 50, 55 and 60, respectively, among men. Again, comparable levels were reached by women at ages 54, 60, and 66 only, i.e. 4, 5, and 6 years later, respectively.

Sensitivity analyses using 5- and 15- rather than 10-year cumulative incidence and mortality in the US as indicators of CRC risk yielded very similar differences in the age at which comparable levels were reached among men and women (differences between 4 and 6 years for 5-year cumulative incidence and mortality, and between 5 and 7 years for 15-year cumulative incidence and mortality).

Analogous calculations for 10-year cumulative CRC mortality in 2001 in 11 large countries from different parts of the world showed very similar age differences between women and men, despite some major variation in the overall levels of CRC mortality (see Table 1). The 10-year cumulative mortality seen among men at age 50 was reached by women between ages 54 and 56 in nine out of 11 countries (median: 55 years); slightly lower and higher ages were only seen for the Russian Federation (52 years), and Japan (57 years), respectively. The 10-year cumulative mortality seen among men at age 55 was reached by women between ages 60 and 62 in the same nine countries (median: 61 years). Again, slightly lower and higher ages were only seen for the Russian Federation (59 years) and Japan (64 years), respectively. The 10-year cumulative mortality seen among men at age 60 was reached by women between ages 66 and 68 in nine countries (median: 67 years) and at ages 69 and 70 in Spain and Japan, respectively.

A time trend analysis on the basis of 10-year cumulative CRC mortality for the US revealed that the sex differences were consistently seen throughout the 25-year period from 1976 to 2001. The sex differences even slightly increased over time.

## DISCUSSION

Our analyses of age- and sex-specific incidence and mortality of CRC in the US and 10 other large countries from different parts of the world indicate that the lower incidence and mortality among women quite consistently translates to an age difference of approximately 4–8 years at which comparable levels of risk are reached. Colorectal cancer incidence and mortality at various ages are closely related to potential benefits of screening, which have to be weighed against costs and potential adverse side effects in choosing the age of screening initiation. Our analysis suggests that the balance in favour of screening is likely to be reached several years later among women than among men. This finding is supported by a recent study from Poland among more than 50 000 participants of a colonoscopy-based screening programme, where prevalence of advanced adenomas was higher at each age among men than among women, prompting the authors to the conclusion that gender-specific CRC-screening recommendations may be warranted (Regula *et al*, 2006). Furthermore, there are indications both from our analysis and from the literature (Fernandez *et al*, 2001) that the gender difference in the epidemiology of CRC has steadily increased during the last few decades. These results may therefore have important implications for the offer of CRC screening programmes and their optimisation in terms of cost effectiveness.

The choice of different, risk adapted ages at initiation of screening is well accepted and established for CRC risk factors other than gender, in particular a history of CRC before age 60 in a first degree relative (Winawer *et al*, 2003; Schmiegel *et al*, 2004; Smith *et al*, 2006). Although the relative risk of CRC among people with such a family history compared to those without is larger than the relative risk of men compared to women (Johns and Houlston, 2001), the prevalence of the former risk factor in the population is much lower than the ‘prevalence’ of male gender. These patterns suggest that appropriate differentiation of age at initiation of CRC screening by gender might be similarly or even more relevant from a public health point of view than the widely practiced differentiation by family history.

Another important epidemiological aspect that might be of importance in the choice of age at initiation of CRC screening is the differential distribution of CRC location among women and men. The proportion of cancer in the distal colon and rectum is considerably lower among women than among men (Stewart *et al*, 1983; Bonithon-Kopp and Benhamiche, 1999; McCashland *et al*, 2001). Therefore, the sex difference in distal CRC occurrence is even larger than the sex difference in overall CRC occurrence. These patterns suggest that age differences may even be more relevant for initiation of screening programmes primarily based on sigmoidoscopy than for screening programmes primarily based on stool tests or colonoscopy.

Epidemiological data on the occurrence of colorectal neoplasms are important, but are not the only factors to be considered in the choice of the age range at which screening is offered. Another important factor is remaining life expectancy (Ko and Sonnenberg, 2005; Lin *et al*, 2006), which is of primary relevance for a potential upper age limit for CRC screening. Given that life expectancy is generally higher among women than among men, there are further reasons to define gender-specific age ranges for CRC screening.

Considering potential gender differences in recommended age ranges for CRC screening, nonepidemiological criteria, such as complexity of guidelines, also have to be taken into account (Lieberman, 2005). One might argue that gender-specific recommendations might add another layer of complexity, which could be a barrier against use of CRC screenings. However, from the patients' point of view, schedules for cancer screening are gender-specific anyway, given that some of the most widely used screening measures refer to female (breast, cervical) and male (prostate) cancers. From the point of view of health-care providers, initiation of CRC screening at different ages for women and men (e.g. 5 years apart) would not seem to be too much of a challenge either.

In the interpretation of our results, the following limitations should be kept in mind. Given the lack of national CRC incidence data for many countries, CRC-mortality data were used along with CRC incidence data in our comparative analyses. As already mentioned, CRC mortality should be a good surrogate parameter given that survival of female and male CRC patients is essentially the same (Bossard *et al*, 2007). In fact, exactly the same age differences by sex were seen for incidence and mortality in our analyses for the US, where both measures were looked at. Another potential limitation is that in some of the countries included in this analysis, some form of CRC screening has already been practiced during the calendar years included in the analyses. This may have affected CRC incidence and mortality to some extent. Furthermore, differential participation in CRC screening among women and men might have affected the gender differences reported in our analysis. However, in the years under investigation, the overall impact of CRC screening is likely to have been limited, and gender differences in screening utilisation quite small compared to the major gender difference in CRC incidence and mortality. In particular, participation in endoscopic screening examinations was slightly lower rather than higher among women compared with men in the US (Seeff *et al*, 2004; Lieberman *et al*, 2005; Meissner *et al*, 2006), and could thus not explain the lower CRC mortality among women.

Our analysis only considers the ‘net differences’ in CRC incidence and mortality between women and men, which might be owing to a variety of reasons. There are suggestions that hormonal effects, both up to menopause and through hormonal replacement therapy (HRT) may protect from or delay development of CRC (Beral *et al*, 2002). To the extent that the recent reduction in HRT use following publication of the results of the Women's Health Initiative Randomised Trial (Rossouw *et al*, 2002; Hersh *et al*, 2004) and the Million Women Study (Beral, 2003; Faber *et al*, 2005) might increase CRC incidence among women, the previous trend of an increasing gender gap in CRC incidence and mortality might be slowed down or possibly reversed.

Our analyses do not allow a general recommendation regarding the best age for initiation of CRC screening. The latter is likely to vary between populations, owing to between-population variation in CRC incidence and mortality, and in CRC screening and treatment costs. Our results suggest, however, that the optimal age for screening initiation is likely to be around 5 years higher for women than for men within populations, assuming that screening is equally effective in women and men. This could imply either postponement of age at initiation of screening among women or advancement of age at initiation among men compared to nongender-specific-screening schemes. It should also be kept in mind that our results pertain to people at average risk of CRC, and they should therefore not be generalised to specific screening strategies for high-risk groups.

In summary, our results suggest that gender specific differentiation of age at initiation of CRC screening by about 5 years might help to utilise screening resources in a more efficient manner. Gender specific screening schedules should therefore deserve careful attention in the design and evaluation of CRC screening programmes.

## Figures and Tables

**Table 1:** Age-adjusted mortality of colorectal cancer among men and women, and ages at which 10-year cumulative mortality among women reaches levels observed among men at ages 50, 55, and 60

		**Age-adjusted mortality^a^**		**Age at which 10-year cumulative mortality among women reaches level observed among men at age**		
**Country**	**Year**	**Men**	**Women**	**50**	**55**	**60**
Australia	2001	17.4	11.2	54	61	67
Canada	2001	14.6	9.6	55	61	67
France	2001	15.8	9.0	56	61	68
Germany	2001	18.2	11.7	56	62	66
Italy	2001	15.1	9.3	54	61	68
Japan	2001	16.4	9.5	57	64	70
Poland	2001	18.7	11.5	55	61	68
Russian Federation	2001	18.7	12.8	52	59	67
Spain	2001	17.6	9.6	55	62	69
United Kingdom	2001	15.9	9.5	55	62	67
United States	2001	13.7	9.5	54	60	66
	1996	15.0	10.3	53	60	67
	1991	16.4	11.2	53	60	66
	1986	17.4	12.3	53	59	65
	1981	18.0	13.0	52	59	65
	1976	18.8	14.5	52	59	65

a Deaths per 100 000 person years, adjusted to the world standard population.
